# Novel Electrocardiographic Patterns for the Prediction of Hypertensive Disorders of Pregnancy—From Pathophysiology to Practical Implications

**DOI:** 10.3390/ijms160818454

**Published:** 2015-08-07

**Authors:** Fabio Angeli, Enrica Angeli, Paolo Verdecchia

**Affiliations:** 1 Division of Cardiology and Cardiovascular Pathophysiology, Hospital “S.M. della Misericordia”, Perugia 06100, Italy; 2 Department of Obstetrics and Gynecology, Hospital “San Giovanni Battista”, Foligno 06034, Italy; E-Mail: angeli.enrica@gmail.com; 3 Department of Internal medicine, Hospital of Assisi, Assisi 06081, Italy; E-Mail: verdec@tin.it

**Keywords:** pregnancy, pregnancy, high-risk, eclampsia, pre-eclampsia, blood pressure, hypertension, pregnancy-induced, electrocardiography

## Abstract

Hypertensive disorders of pregnancy are a major cause of poor outcome, including placental abruption, organ failure, cerebrovascular accident and disseminated intravascular coagulation. These disorders are associated with increased fetal risk of intrauterine growth restriction, intrauterine death and prematurity. Electrocardiography (ECG) recently emerged as a useful tool to evaluate cardiovascular complications during pregnancy. Specifically, left atrial abnormalities detected by standard ECG are associated with a fourfold increased risk of developing hypertensive disorders during pregnancy. The mechanisms linking left atrial abnormality on ECG with hypertensive disorders are still elusive. Several mechanisms, possibly reflected by abnormal left atrial activation on ECG, has been suggested. These include increased reactivity to angiotensin II and up-regulation of angiotensin type 1 receptors, with activation of autoantibodies targeting these receptors.

## 1. Introduction

Hypertension disorders complicate approximately 6%–11% of all pregnancies and remain leading causes of maternal and perinatal morbidity and mortality, particularly when elevated blood pressure (BP) is due to preeclampsia, either alone or superimposed on chronic vascular disease [1,2,3].

Specifically, they rank among the leading causes of maternal adverse outcome, along with embolism, hemorrhage and non-obstetric injuries, accounting for almost 15% of such deaths [2].

They also contribute significantly to stillbirths and fetal complications including abruptio placentae, intrauterine growth restriction, premature delivery, and intrauterine fetal death [4].

Hypertension in pregnancy is defined by office systolic BP ≥ 140 mmHg and/or diastolic BP ≥ 90 mmHg. According to current guidelines [5], hypertensive disorders during pregnancy are classified into four categories:

(i) Pre-existing or chronic hypertension (hypertension that was present before pregnancy or that develops at <20th week gestation);
(ii) Gestational hypertension (hypertension that develops for the first time at ≥20th week’ gestation; this definition is preferred over the older term of pregnancy-induced hypertension);
(iii) Preeclampsia-eclampsia;
(iv) Other hypertensive effects (including transient hypertensive effect, white-coat hypertensive effect and masked hypertensive effect).

For pre-existing and gestational hypertension, there are two subgroups: (1) with comorbid conditions that mandate tighter BP control as outside pregnancy (to protect end-organ function); and (2) with preeclampsia (given its substantial maternal and perinatal risks) [5].

Approximately 1% of pregnancies are complicated by pre-existing hypertension, 5%–6% by gestational hypertension, and 1%–2% by preeclampsia [5]. Nevertheless, rates of hypertensive disorders of pregnancy are anticipated to rise given older and more obese obstetric populations with more antecedent medical complications [5].

Epidemiological evidences supporting the worse prognosis associated with hypertension in pregnancy provide a strong basis for developing risk prediction models to identify women whose gestations may be considered at high risk for hypertensive disorders. These women may require a closer surveillance and preventive treatments [4].

Of note, of the many risk markers for hypertensive disorders [6,7,8,9,10,11,12,13,14], some are known at booking and increase the risk of hypertensive disorders two- to fourfold [15]. They include pre-existing hypertension, diabetes mellitus and renal disease, previous preeclampsia, antiphospholipid antibody syndrome, overweight/obesity, inter-pregnancy interval ≥10 years, and multiple pregnancy [15,16].

Just recently, the additive value of some instrumental techniques (including uterine artery Doppler velocimetry, electrocardiography [ECG] and ambulatory BP monitoring) and their combinations with maternal factors and biochemical markers to refine risk stratification for hypertensive disorders in pregnancy has also been evaluated [17,18,19,20]. In this context, some observations suggested that abnormal ECG patterns may increase the risk for hypertensive disorders of pregnancy [16,17,21].

We aimed to specifically address the evidence provided in this regard. We also critically discussed the available data supporting the concept that specific ECG patterns occurring in the first trimester of pregnancy may have clinical relevance for the risk prediction of hypertensive disorders.

For this purpose, we searched for clinical studies using research Methodology Filters [22,23]. The following research terms were used: “pregnancy”, “pregnancy, high-risk”, “eclampsia”, “pre-eclampsia” “blood pressure”, “hypertension, pregnancy-induced” and “electrocardiography”. We also checked the reference list of identified articles and previous systematic reviews to find other relevant studies.

## 2. Hemodynamic Changes in Pregnancy

Knowledge of cardiovascular adaptations in pregnancy is required to correctly interpret ECG in the gravida, to predict the effects of pregnancy on the woman with underlying cardiac disease, and to understand how the fetus will be affected by maternal cardiac disorders.

**Figure 1:**
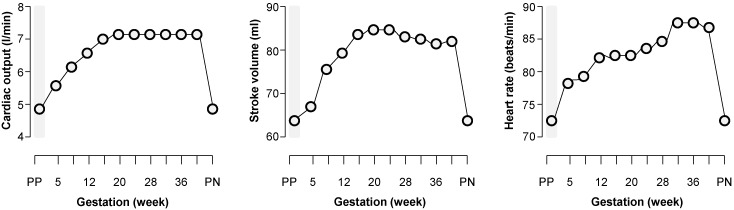
Changes in cardiac output from the non-pregnancy state throughout pregnancy. Related to the increase in plasma volume and vasodilation, cardiac output increases by 30%–50% during pregnancy via increases in both stroke volume and heart rate. PP = pre-pregnancy state; PN = post-natal state. Data from ref. [25].

The major hemodynamic changes induced by pregnancy include an increase in cardiac output (Figure 1), sodium and water retention leading to blood volume expansion, and reductions in systemic vascular resistance and systemic BP [24,25]. These changes begin early in pregnancy, reach their peak during the second trimester, and then remain relatively constant until delivery [24] (Figure 1). They contribute to optimal growth and development of the fetus and help to protect the mother from the risks of delivery, such as hemorrhage [24,25].

Specifically, during pregnancy there is a 30%–50% increase in the extracellular fluid and a 30%–40% increase in plasma volume. The driving force for the increase in extracellular volume is decrease in the systemic vascular resistance, as reflected by a fall in the systolic and diastolic BP in early gestation [25].

Vasodilating factors such as nitric oxide (NO) play an important role in the decrease in vascular resistance [26]. The generalized vasodilation results in a compensatory activation of the renin-angiotensin-aldosterone system (RAAS), leading to water and sodium retention [27].

Furthermore, the renal blood flow and glomerular filtration rate markedly increase during pregnancy, and peak at approximately 50% above non-pregnant levels in the second trimester [28].

Hemodynamic investigations in pregnant women with hypertensive disorders are scanty. Generally, in hypertensive disorders of pregnancy, the expansion of plasma volume and the decrease in vascular resistance are less pronounced [29]. As a direct consequence of the decreased plasma volume, cardiac output is also lower than in normal pregnancy [30]. Moreover, uterine and umbilical artery Doppler flow measurements have revealed that pregnancies complicated either by pre-eclampsia or growth restriction are characterized by a compromised uteroplacental flow, suggesting that the normally occurring expansion of plasma volume and increase in cardiac output are essential for the maintenance of a sufficient uteroplacental flow [29].

More recently, the picture has been complicated by different definitions and classifications of severe hypertensive disorders in pregnancy. Of note, different classifications of preeclampsia (mild, moderate and severe, as well as early and late) have been proposed [31,32].

Nevertheless, the concept of early (before 34 weeks) and late (after 34 weeks) preeclampsia is more modern and widely accepted for the evidence that these two entities probably recognize different etiologies and develop through different models of cardiovascular maternal adaptations [33,34,35]. Specifically, early preeclampsia, as a placenta-mediated disease [36,37], is commonly associated with abnormal uterine artery Doppler, fetal growth restriction, and adverse maternal and neonatal outcomes [33,34,35]. In contrast, late-onset preeclampsia is mostly associated with normal or slight increased uterine resistance index, a low rate of fetal involvement, and more favorable perinatal outcomes [33,34,35].

Maternal echocardiography may be useful to identify at 24 weeks of gestation women at increased risk of early complications through the evaluation of maternal hemodynamics and left ventricular geometry. High maternal total vascular resistance, increased relative wall thickness of the left ventricle, small left ventricular diameter and development of concentric left ventricular hypertrophy have been related to early-onset preeclampsia, suggesting an involvement of the whole cardiovascular system [38,39,40]. On the other hand, patients with late forms of preeclampsia showed low total vascular resistance, high cardiac output, increased diameter of the left ventricle with an intermediate relative wall thickness (eccentric geometry) as compared to controls and early preeclampsia [38,39,40,41].

## 3. Pregnancy-Induced ECG Changes

**Figure 2:**
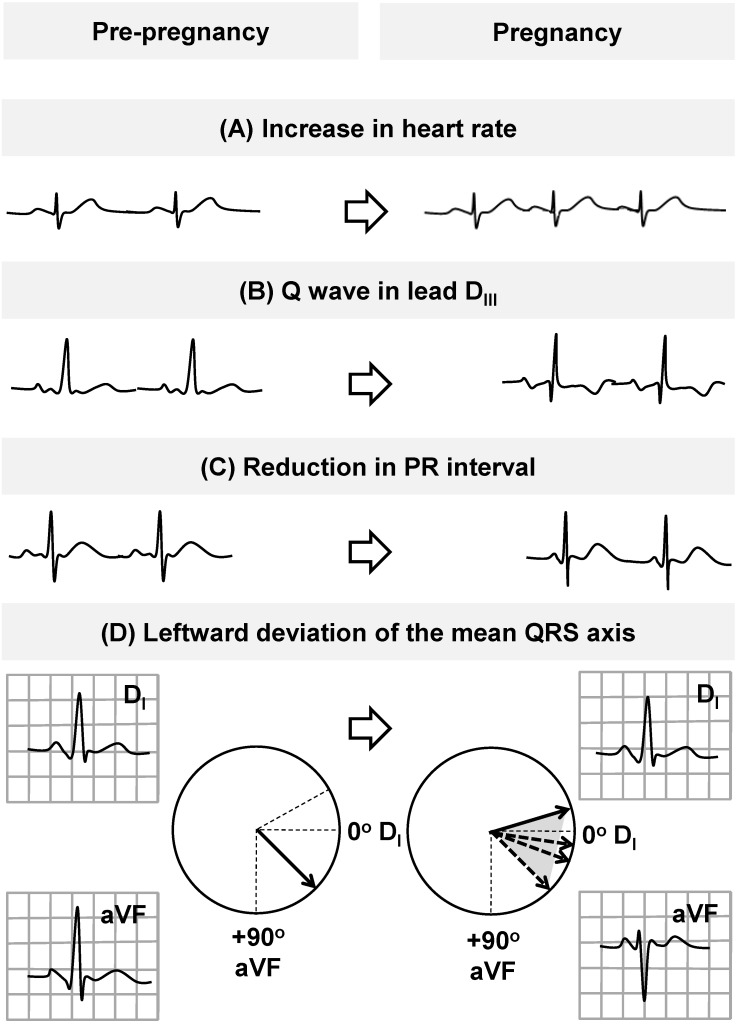
Normal electrocardiographic (ECG) adaptations during pregnancy. ECG changes in normal pregnancy include a reduction in the mean values of PR interval (**C**), sinus tachycardia (**A**), left axis deviation (**D**), inverted or flattened *T*-waves, and a *Q*-wave in lead D_III_ (**B**) [21].

Pregnancy affects the ECG at some time point and there is restoration of these pregnancy-induced changes late in pregnancy or following delivery (Figure 2) [21,42,43,44,45,46,47,48,49].

Heart rate (HR) increases progressively throughout the pregnancy, reaching a peak during the third trimester. This increase in HR seems related to hormonal factors in early stages of pregnancy and later to increased left atrial diameter and sympathetic activation (sinus-node remodeling) [21]. Gestational age also impacts QRS complex and *T*-waves, promoting a leftward axis shift as pregnancy progresses. In particular, a leftward deviation of the mean QRS axis during the second and third trimesters of pregnancy and then rightward before delivery is observed in the majority of women [21,42,43,44,45,46,47,48,49]. PR interval exhibits a significant reduction in the mean values during pregnancy, while the QRS amplitude generally increases slightly in the late pregnancy (but without a clear evidence of left ventricular hypertrophy). No clinically significant changes occur in other ECG intervals (including QT interval) or cardiac rhythm (Figure 2) [21,42,43,44,45,46,47,48,49].

## 4. ECG Features of Increased Risk

Only a few studies have investigated the ECG changes in pregnant women with hypertensive disorders during normal pregnancy [21]. Nevertheless, there is evidence that hypertensive disorders of pregnancy can be associated with changes in *P*-wave morphology and QT interval (Table 1) [21].

**Table 1:** Main clinical studies which demonstrated electrocardiographic features of increase risk for hypertensive disorders of pregnancy.

Study	[Ref.]/Year	Design	Sample Size (*n*)	ECG Features
Isezuo and Ekele	[50]/2004	Prospective	60	QT interval
Baumert *et al.*	[51]/2010	Case-control	64	QT interval
Angeli *et al.*	[17]/2011	Prospective	221	*P*-wave morphology
Raffaelli *et al.*	[52]/2014	Case-control	152	QT interval, QT dispersion and *P*-wave duration

### 4.1. QT Interval

QT interval seems to be unaffected by normal pregnancy [42]. Conversely, pregnancies with abnormal uterine perfusion that developed pathological outcomes seem to be paralleled by changes in ventricular repolarization that may precede clinical symptoms [51].

In a recent study by Baumert and co-workers [51], monthly ECGs were recorded in 32 pregnant women with normal uterine perfusion and 32 pregnant women with abnormal perfusion, starting from the 20th week of gestation until 3 days postpartum. Among pregnancies with abnormal uterine perfusion, 17 pregnancies developed preeclampsia and/or small-for-gestational-age infants. While among patients with normal perfusion the QTc interval was unaltered, pregnancies with abnormal uterine perfusion that developed pathological outcomes showed a trend towards shorter QTc. Specifically, the QTc interval of women with pathological pregnancy outcomes appeared to shorten progressively throughout the second half of gestation, before the onset of clinical symptoms of hypertension or proteinuria [51].

More recently, Raffaelli and co-workers [52] investigated the effect of pre-eclampsia on electrical cardiac activity and demonstrated that pregnancies complicated by pre-eclampsia had a significant alteration of ventricular repolarization. They compared pre-partum ECGs of 76 consecutive pre-eclamptic women with those of 76 healthy pregnant women. All of the routine ECG parameters were considered, and ventricular repolarization was assessed by QT interval and QT dispersion (QTd).

The routine evaluation of the ECGs in both groups was mostly non-pathological. However, pre-eclamptic women showed a lower HR (77.4 ± 14.3 *vs.* 81.6 ± 11.0 beats per minute [bpm]), a longer mean QTc interval (442.7 ± 26.7 *vs.* 423.7 ± 20.7 ms) and a higher QTd (24.0 *vs.* 22.0 ms) than the control group [52].

In this context, it is worthy to be mentioned that QTd is an indicator of inhomogeneity in ventricular activity and its prolongation is correlated with an increased incidence of ventricular arrhythmias and is a predictor of all-cause mortality [53]. Thus, prolonged QT dispersion could explain the increased incidence of serious ventricular arrhythmias and adverse outcomes in pregnant women with pre-eclampsia [52].

Similarly, in a prospective study by Isezuo and Ekele [50], eclampsia was associated with prolonged ventricular repolarization. Briefly, the QT interval corrected for HR, serum calcium, magnesium and potassium were compared among 30 intrapartum eclamptics and 30 age, parity and gestational age-matched women with uncomplicated pregnancy. HR ranged from 76 to 163 bpm and 65 to 112 bpm among patients and controls, respectively. The corresponding QTc were 390–572 ms and 390–460 ms, respectively. Compared to the controls, the eclamptic patients had higher frequency of abnormal QTc (46.7% *vs.* 6.6%, odds ratio [OR]: 9.2; 95% confidence interval [CI]: 1.61–68.48, *p* = 0.01) as measured on the surface ECG [50].

### 4.2. Abnormal P-Wave Morphology

Studies by the group above [52] and our [16,17] investigated abnormal *P*-wave morphology during pregnancy as potential predictor of hypertensive disorders during pregnancy.

In the recent analysis by Raffaelli *et al.* [52], *P*-wave duration was significantly longer in the pre-eclamptic women than in the control group of normal pregnancy [52].

Furthermore, a prospective collaborative screening study between gynecologists, internists and cardiologists evaluated the potential additive role of standard ECG in the identification of women at increased risk for hypertensive complications [17].

**Figure 3:**
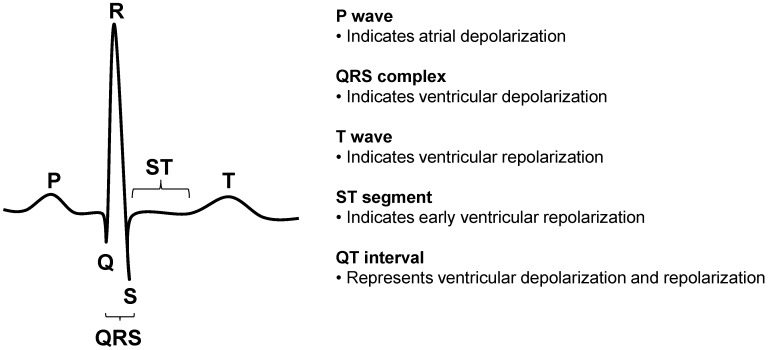
ECG components and intervals tested as predictors of hypertensive disorders of pregnancy in nulliparous healthy women with singleton pregnancies.

At the first antenatal visit, 12-lead ECG was recorded and the following ECG parameters were analyzed (Figure 3): HR, QRS duration, QTc interval, Cornell voltage, ST-T abnormalities, and left atrial abnormality.

The QT interval was measured as the time between the start of the *Q*-wave and the end of the *T*-wave and corrected by HR according to the Bazett’s formula [54].

The Cornell voltage was computed as the sum of the amplitudes of *S*-wave in V_3_ and *R*-wave in aVL [55].

ST-T changes were analyzed according to the Minnesota Coding [56]. Criteria for ST-T changes were any of the following: (1) coexistence, in any leads I, II, aVL or V3–V6 of ST-segment horizontal or downward sloping depression ≥0.05 mV (code 4–1 or 4–2) plus *T*-wave asymmetric inversion (code 5–1 or 5–2); (2) ST-J depression <0.05 mV with ST-segment downward sloping and segment or *T*-wave nadir >0.05 mV below P-R baseline, in any of leads I, II, aVL or V2–V6 (code 4–3); (3) ST-J depression of ≥0.10 mV and ST-segment upward sloping or *U*-shaped, in any of leads I, II, aVL or V2–V6 (code 4–4); (4) *T*-wave amplitude zero (flat), negative or diphasic (negative-positive type only) with <0.10 mV negative phase in lead I, II, V3–V6, or in lead aVL when *R*-wave amplitude is ≥0.5 mV (code 5–3); (5) *T*-wave amplitude positive and *T*- to *R*-wave amplitude ratio <1:20 in any of leads I, II, aVL or V3–V6 when *R*-wave amplitude in the corresponding leads is ≥1.0 mV (code 5–4).

*P*-wave morphology was analyzed in all of the standard leads. The criteria used for the diagnosis of *P*-wave abnormality in lead V_1_ were: (1) bipeak interval in deeply notched *P*-wave with (2) terminal forces equal to or more negative than −0.04 mm·s, as obtained from the product of the depth of the terminal negative deflection and its duration [57,58]. The following other criteria were used for the diagnosis of LA abnormality in any other lead than V_1_: (1) bipeak interval in deeply notched *P*-waves wider than 0.04 s; or (2) *P*-wave/PR-segment ratio greater than 1.6; or (3) *P*-wave higher than 3 mm; or (4) total *P*-wave duration greater than 0.11 s [57,58].

The primary outcome of the study was the development of gestational hypertension, pre-eclampsia and eclampsia. The secondary outcome was a composite measure of hypertensive disorders and other pregnancy complications including fetal growth restriction, HELLP (hemolysis, elevated liver enzymes, low platelets) syndrome, placental abruption, stillbirth, premature delivery and neonatal death [4,59,60].

Overall, 308 women were screened and 87 excluded. Thus, a total of 221 pregnant women were included in the final analysis [17].

The primary outcome of hypertensive disorders occurred in 28 women. Specifically, gestational hypertension occurred in 22 women, 5 women experienced pre-eclampsia (3 of these developed HELLP syndrome) and 1 woman had eclampsia. Among pre-eclamptic women, 3 had late-onset pre-eclampsia (≥34 weeks gestation).

The secondary composite outcome was recorded in 43 women. Multiple events (hypertensive disorders and other maternal or fetal/neonatal complications) were observed in 9 women.

Overall, premature deliveries occurred in 14 women, 6 women delivered growth-restricted neonates, 2 women experienced placental abruption and 2 congenital heart defect requiring admission to neonatal nursery were recorded. No neonatal deaths were observed.

Study population was sub-divided into two groups by occurrence of hypertensive disorders during pregnancy. At entry, women with development of hypertensive disorders differed under some aspects from those who did not experience these events: Weight, body mass index (BMI) and BP were higher in women with hypertensive diseases than in those without (all *p* < 0.05).

Left atrial abnormality in lead V_1_ was also more prevalent in women with hypertension disorders (*p* = 0.002). Age, laboratory tests, HR and other ECG parameters including left atrial abnormality observed in other leads than V_1_ did not differ between the two groups.

In a multivariable model, mean arterial pressure (MAP) and left atrial abnormality in lead V_1_ were independent predictors of hypertensive disorders.

In particular, the presence of LA abnormality in lead V_1_ was associated to a 4-fold increased risk of developing hypertensive disorders (OR: 4.35; 95% CI: 1.84–10.31; *p* = 0.001).

The final predictive model discriminated well between women who developed hypertensive disorders and women who remained normotensive during pregnancy, with an area under the curve of 0.754 (95% CI: 0.667–0.841, *p* < 0.0001).

**Figure 4:**
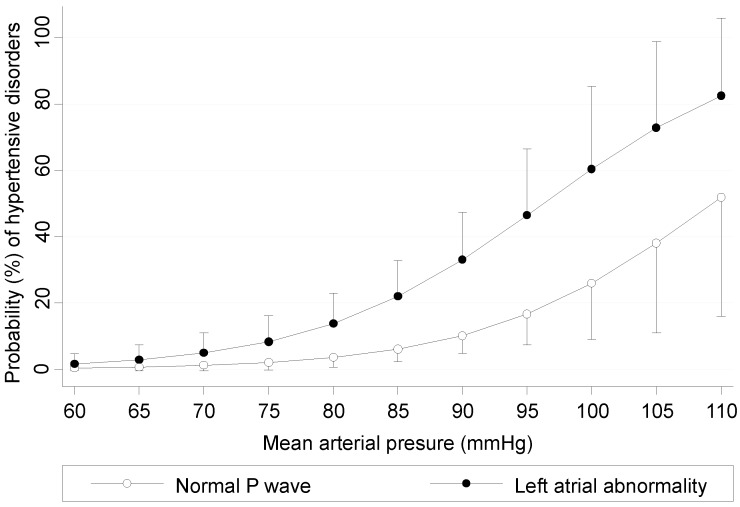
Risk of developing hypertensive disorders (probability with 95% confidence intervals) according to baseline mean arterial pressure and *P*-wave morphology in lead V_1_.

The probability of developing hypertensive disorders according to baseline MAP and *P*-wave morphology (normal vs left atrial abnormality in lead V_1_) is depicted in Figure 4.

Although the primary outcome of the study was the development of hypertensive disorders, the same prediction model also proved significance to identify pregnant women at increased risk for the occurrence of maternal and fetal/neonatal complications [17].

## 5. *P*-Wave Morphology and Risk of Hypertensive Disorders: Mechanisms

Abnormality of *P*-wave morphology in lead V_1_ tested as predictor of hypertensive disorders during pregnancy [17] is commonly used as an ECG sign of left atrial enlargement and it may be easily diagnosed by traditional visual interpretation of ECG tracings, without any need of digitalization or other computer facilities [16].

Left atrial enlargement is very common in hypertensive subjects and may be an early sign of heart involvement in arterial systemic hypertension [61,62].

In this context, some echocardiographic studies showed that women developing hypertensive complications during pregnancy may have different echocardiographic patterns than controls [38,40,41,63].

Nevertheless, the potential link between left atrial abnormality detected early in pregnancy by echocardiography or ECG and development of hypertensive disorders remains elusive.

**Figure 5:**
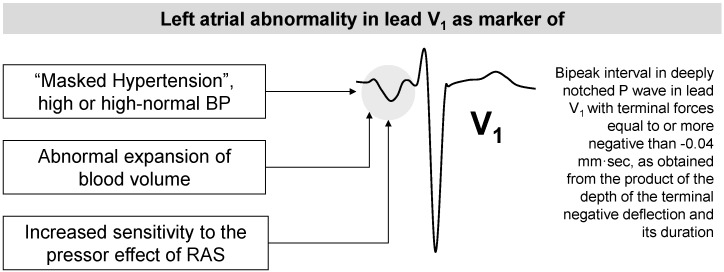
Potential mechanisms linking left atrial abnormality detected early in pregnancy by ECG and development of hypertensive disorders during pregnancy. Data from ref. [16,17,21].

Some possible mechanisms are supported by epidemiological data and experimental models [16,17,21]. As depicted in Figure 5, they include hemodynamic and molecular mechanisms.

### 5.1. Hemodynamic Mechanisms

Many clinical studies in hypertensive patients found a significant relation between BP overload and left atrial abnormalities [61,62,64,65,66]. The lack of association between left atrial enlargement at ECG and BP values observed in our study [17] supports the poor ability of casual BP measurements obtained at the time of the first visit to the hospital to detect the real BP load of pregnant women. In other words, the tendency of BP to decrease in early pregnancy [67] may mask the real load of women with abnormal BP before pregnancy. Thus, the presence of an abnormal *P*-wave in the early phase of gestation may be considered as a marker of masked hypertension or high or high-normal BP values.

Expansion of blood volume directly affects left atrial pressure and dimensions [68] with a consequent increased release of atrial natriuretic peptide (ANP) [69]. As described above, the major hemodynamic changes during pregnancy include an increase in cardiac output, sodium and water retention leading to blood volume expansion. These changes begin early in pregnancy, reach their peak during the second trimester and then remain relatively constant until delivery [70,71]. In uncomplicated pregnancy, plasma renin activity tends to be increased and ANP levels are slightly reduced [72,73]. In contrast, plasma ANP concentrations have been extensively reported as increased in patients with hypertensive disorders during pregnancy, especially in patients with severe pre-eclampsia [74,75,76]. The detection of left atrial enlargement may be a marker of an abnormal expansion of blood volume in the early phase of pregnancy which may affect left atrial morphology [68] and the possible consequent increased release of ANP may predispose to the development of hypertensive disease.

Moreover, left ventricular hypertrophy has been suggested to mediate, at least partially, the relation between hypertension and left atrial enlargement [77].

The presence of abnormal *P*-wave morphology at ECG may be a marker of “latent” abnormal left ventricular mass (hypertrophized ventricle) which commonly develop in both early and late onset preeclampsia (as manifestation of an underfilling state with pressure overload and an overfilling state without pressure overload, respectively) [38,39,40,41].

Nevertheless, echocardiographic data on the relationship between the simultaneous adaptations of left ventricle and left atrium during hypertensive disorders of pregnancy are scarce and conflicting [78,79,80]. In a recent case-control study involving 37 pregnant women (20 with pre-eclampsia and 17 age-matched controls), ventricular septal thickness, posterior wall thickness, and left ventricular mass index were significantly greater in patients with preeclampsia than in controls (*p* < 0.0001) [80]. Nevertheless, there were no significant differences in left atrial diameter [78]. Similar results were also obtained by Borghi *et al.* [79] in a sample of 75 pregnant women. Conversely, Vazquez *et al.* failed to demonstrate changes of both left ventricle and left atrium in women with preeclampsia [80].

Finally, it is worthy to be mentioned that diastolic dysfunction contributes to left atrial remodeling and that atrial enlargement has been suggested as a marker of the severity and duration of diastolic dysfunction [81]. Diastolic cardiac function varies during pregnancy. A relationship between preload, afterload, morphologic, and diastolic function modifications appears to exist as a consequence of the hemodynamic modifications which occur during physiologic pregnancy [82]. Diastolic function analysis may be useful to identify women who fully adapt to pregnancy, and to understand the mechanisms that might be involved in pregnant women who develop hypertensive disorders [82].

Of note, a prospective study including 1688 high risk nulliparous women demonstrated that diastolic function differs among women with preeclampsia and controls [41].

In particular, some Doppler values (E/A ratio and *A*-wave velocity) suggested a more altered diastole in both early and late preeclampsia compared to controls 1 year postpartum [41].

### 5.2. Molecular Mechanisms

**Figure 6:**
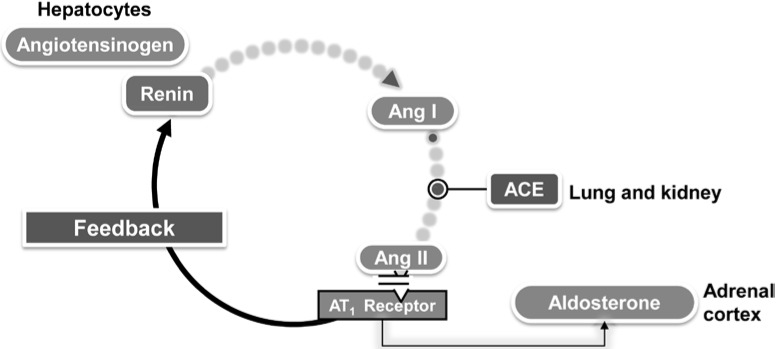
Schematic of the classic RAAS system cascade. The biosynthesis of renin by the juxtaglomerular cells is a key determinant of the activity of the RAS. Renin regulates the initial, rate-limiting step of the RAS by cleaving the *N*-terminal portion of a large molecular weight globulin, angiotensinogen, to form the biologically inert decapeptide Angiotensin I or Angiotensin-(1–10). The inactive decapeptide Angiotensin I is hydrolyzed by angiotensin-converting enzyme (ACE), which removes the *C*-terminal dipeptide to form the octapeptide Angiotensin II [Ang-(1–8)], a biologically active, potent vasoconstrictor.

Other changes during pregnancy include a marked stimulation of the all measured elements of the RAAS [60,83,84,85] (Figure 6). There is an early increase in renin due to extra-renal local release by the ovaries and maternal decidua [83,84,85]. Angiotensinogen synthesis by the liver is increased by circulating estrogen produced by the growing placenta [83,84,85]. This leads to increased serum Angiotensin II and aldosterone levels [83,84,85].

Although Angiotensin II levels increase during pregnancy, normotensive pregnant women are actually refractory to its vasopressor effects.

Assali *et al.* [86] demonstrated that the pregnant woman requires twice as much Angiotensin II intravenous infusion over a non-pregnant woman to achieve the same vasomotor response. This is thought to be due to the presence of increased progesterone and prostacyclins which can decrease Angiotensin II sensitivity [87].

Additionally, in normal pregnancy Angiotensin Type 1 (AT_1_) receptors are monomeric and are inactivated by reactive oxygen species (ROS). This is in comparison to the heterodimeric state seen in Angiotensin II sensitive conditions [88].

Taken together, these observations help explain why a greater Angiotensin II stimulus is required in order to achieve the appropriate vasomotor response in normotensive pregnancies.

Conversely, in preeclamptic women the circulating levels of renin, Angiotensin I and aldosterone are lower than their normotensive counterparts [85]. There are two exceptions to the decreases observed. The ACE level is approximately the same and Angiotensin 1–7, a vasodilatory member of the RAAS, is significantly reduced in preeclampsia [85].

Moreover, in pregnant women developing severe forms of hypertensive disorders there is a loss of the normal pregnancy-associated refractoriness to pressor agents and the sensitivity to infused Angiotensin II increases weeks before overt disease [89,90,91,92,93,94,95,96,97].

Explanations for the increased reactivity to Angiotensin II include up-regulation of receptor sensitivity, synergy with circulating autoantibodies agonistic to the AT_1_ receptor and decreases in the level of circulating Angiotensin 1–7 [89,90,91,92,93,94,95,96,97].

**Figure 7:**
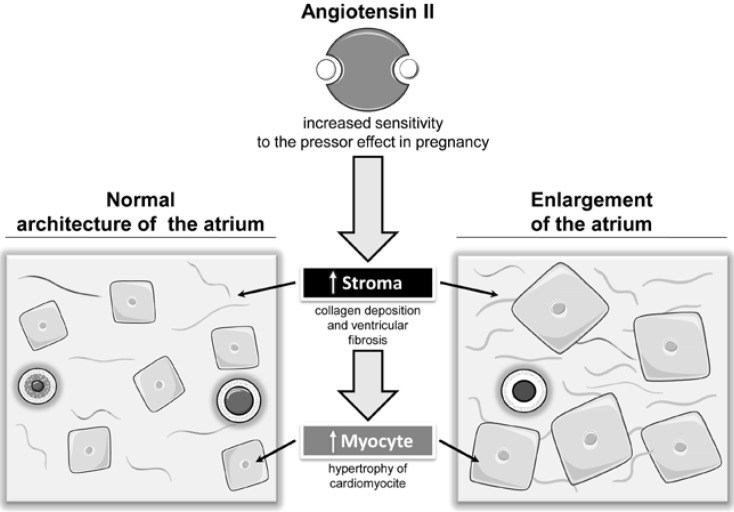
Effects on left atrium of Angiotensin II in pregnancy complicated by hypertensive disorder.

Experimental studies have shown that the RAAS in the heart could trigger cardiac hypertrophy [98] and a massive enlargement of the atria caused by myocyte hyperplasia [99]. Thus, an increased sensitivity to the pressor effect of the Angiotensin II in some women may be involved in the development of ECG signs of left atrial enlargement and may predispose to the development of hypertensive disorders [16,17,21] (Figure 7).

## 6. Conclusions and Perspectives

Several risk factors have been proposed as potential predictors of hypertensive disorders during pregnancy [11]. They include maternal demographics and clinical characteristics [15], laboratory markers (plaminogen activator inhibitor, placental growth factor, von Willerand factor, *C*-reactive protein and serum uric acid), provocative biophysical tests (angiotensin II challenge test, roll-over test, isometric exercise test), urinary proteomics, inherited thrombophilias (factor V Leiden mutation, prothrombin gene mutation, protein C or S deficiency and antithrombin III deficiency), antiphospholipid antibodies and abnormal maternal serum markers (alpha fetoprotein, human chorionic gonadotrophin and plasma tumor necrosis factor alpha) [100,101,102,103,104,105,106,107,108].

Nevertheless, early prediction of hypertensive disorders in healthy and initially normotensive pregnant women remains problematic, partly because severe forms such as preeclampsia and eclampsia are etiologically complex and heterogeneous conditions [16].

No guidelines exist for an appropriate and cost-effectiveness screening and early detection of hypertensive disorders in the community and there is no uniformity in referral thresholds and assessment procedures. In addition, systematic reviews suggest that any single test may lack predictive value or practical utility to be applied at large [100,101,102].

In the last few years, some clinical studies tested the combinations of different risk markers to develop multivariable models for the prediction of hypertensive disorders during pregnancy [8,15,16,17]. The use of multiple markers in a screening approach may reflects different aspects of the hypertensive disease process and increases the specificity and sensitivity of the screening [17].

In this context, some studies analyzed the additive value of ECG and its combination with maternal factors and biochemical markers to refine risk stratification for hypertensive disorders in pregnancy [8,15,16,17].

In particular, changes during pregnancy in QT interval and *P*-wave morphology have been tested as potential features of increased risk for the development of hypertensive disorders.

With regard to QT interval changes and its time course during gestation, clinical studies have produced conflicting results and it is not clear whether alterations in the QT interval precede clinical manifestations of hypertensive disorders [50,51,52].

Moreover, as documented by the analysis by Raffaelli and co-workers [52], the prolonged QTc interval observed in the acute phase of hypertensive disorders of pregnancy might be only the result of high-dose administration of MgSO_4_. Thus, in our opinion the notion that increased ventricular repolarization heterogeneity (as documented by higher values of QTd) is a spontaneous high-risk feature of pre-eclampsia for the development of adverse events remains questionable [21].

More interestingly, a recent prospective screening study in nulliparous healthy women with singleton pregnancies showed that combination of left atrial enlargement at ECG and BP measurement improves the accuracy of risk models for the prediction of hypertensive disorders of pregnancy, especially for the more severe forms [17].

In conclusion, there is no single screening test that may be definitely assumed as reliable and cost-effective for the prediction of hypertensive disorders in pregnancy. Most of the tested markers suffer from poor sensitivity and poor positive predictive values, and the majority of them are not suitable for routine use in clinical practice [16].

However, recent studies have generated the hope that combining new screening tests with risk markers will provide the sensitivities and likelihood ratios required for prediction of hypertensive disorders in pregnancy enhancing the ability of clinicians to detect subgroups of pregnant women at increased risk.
